# Barriers and Facilitators of Pharmacoeconomic Studies: A Review of Evidence from the Middle Eastern Countries

**DOI:** 10.3390/ijerph19137862

**Published:** 2022-06-27

**Authors:** Abdulaziz Ibrahim Alzarea, Yusra Habib Khan, Abdullah Salah Alanazi, Muhammad Hammad Butt, Ziyad Saeed Almalki, Abdullah K. AlAhmari, Saud Alsahali, Tauqeer Hussain Mallhi

**Affiliations:** 1 Department of Clinical Pharmacy, College of Pharmacy, Jouf University, Sakaka 72388, Saudi Arabia; aizarea@ju.edu.sa (A.I.A.); asdalananzi@ju.edu.sa (A.S.A.); 2 Health Sciences Research Unit, Jouf University, Sakaka 72388, Saudi Arabia; 3 Faculty of Pharmacy, University of Central Punjab, Lahore 54000, Pakistan; hmdbut@ucp.edu.pk; 4 Department of Clinical Pharmacy, College of Pharmacy, Prince Sattam Bin Abdulaziz University, Al-Kharj 11942, Saudi Arabia; z.almalki@psau.edu.sa (Z.S.A.); a.alahmari@psau.edu.sa (A.K.A.); 5 Department of Pharmacy Practice, Unaizah College of Pharmacy, Qassim University, Buraydah 52571, Saudi Arabia; s.alsahali@qu.edu.sa

**Keywords:** pharmacoeconomics, barriers, facilitators, Middle East, implementation of pharmacoeconomics, economic evaluation

## Abstract

The world is facing a continuous increase in medical costs. Due to the surge in disease prevalence, medical science is becoming more sensitive to the economic impact of medications and drug therapies. This brings about the importance of pharmacoeconomics, which is concerned with the effective use of health resources to optimize the efficiency and costs of medications of treatment for the best outcomes. This review was conducted to find out the potential barriers and facilitators to implementing pharmacoeconomic studies in the Middle Eastern region having both high- and low-income countries. The varying economies in the region depict diverse healthcare systems where implementation of pharmacoeconomics faces a large number of challenges and is also aided by numerous facilitators that contribute to the growth of its implementation. In this context, we have reviewed the status of pharmacoeconomics in Middle Eastern countries in research databases (Google Scholar, MEDLINE, Science Direct and Scopus) using keywords (“pharmacoeconomics”, “barriers”, “facilitators”, “Middle East”). The study reported that Yemen, Syria, Palestine, Iran, Iraq, Jordan and Lebanon are the lowest-income countries in the Middle East and the implementation of pharmacoeconomics is the poorest in these states. The UAE, Saudi Arabia and Israel are high-income rich states where economic aspects were comparatively better but still a large number of barriers hinder the way to its effective implementation. These include the absence of national governing bodies, the lack of data on the effectiveness of medications, the absence of sufficient pharmacoeconomic experts and the lack of awareness of the importance of pharmacoeconomics. The main facilitators were the availability of pharmacoeconomic guidelines, the encouragement of pharmacoeconomic experts and the promotion of group discussions and collaborations between researchers and policymakers. Cost-benefit analysis is still evolving in Middle Eastern countries, and there is a great need for improvement so that states can effectively benefit from cost analysis tools and utilize their health resources. In this regard, governments should develop national governing bodies to evaluate, implement pharmacoeconomics at the local and state levels and bring about innovation in the field through further research and development incorporating all sectors of pharmacy and pharmaceutics. The data presented in this research can further be extended in future studies to cover the various domains of pharmacoeconomics including cost-minimization analysis, cost-effectiveness analysis and cost-benefit analysis and their applications within the healthcare sectors of Middle Eastern countries.

## 1. Introduction

With the rise in global healthcare expenditures, the pharmaceutical and medical community has become sensitive to medication costs and evaluation. The assessment of available healthcare services and goods extends way beyond just their safety evaluation and encompasses their monetary impact on healthcare finances and cost considerations. To use medical resources wisely, it is imperative to implement economic efficiency by reasonable use of pharmacoeconomic analysis and strategies for their implementation [1].

Pharmaceutical analysts and practitioners have made cost analysis studies an emerging discipline of crucial importance. It covers the medical world’s financial and economic considerations, aiming to evaluate the costs and consequences of medical products and services on individual patients, overall healthcare systems and pharmaceutical industries. The boundless use of healthcare equipment due to the rise in the prevalence of diseases calls for a need for more investment in providing this equipment, which puts a toll on the healthcare budget of states in general and pharmaceutical companies in particular. The challenge of providing quality healthcare with minimum costs brings the importance of pharmacoeconomics to the surface [2].

How much drug therapy costs and what are its eventual benefits concerning those costs is the basic working principle of pharmacoeconomics. The evaluation of such principles provides an insight on how to reasonably allocate and utilize budgetary resources in the healthcare system. This is why this aspect of pharmaceutical science is essential. If used wisely, it can significantly help combat the financial problems of the healthcare system by optimizing cost–benefit relationships. In addition, it helps in the decision making for health-related policies and eases the economic evaluation process for clinical trials of new medical products. Economic evaluation under the flag of pharmacoeconomics can be carried out in four ways: cost-utility analysis, cost-minimization analysis, cost-benefit analysis and cost-effectiveness analysis. The type of analysis chosen depends on the context in which it is performed and the specific outcome required for that context [3].

Health care costs have seen an increase with the recent COVID-19 pandemic [4]. According to the report of the IMS Institute for Healthcare Informatics, approximately 500 billion standard units of drugs were consumed by the population of countries of the Middle East and North Africa region in 2020 [5]. However, there is a strong association between inefficient pharmaceutical spending on the part of the public and a dire need for drugs and medicines. In this case, families with a very low monthly income are completely dependent on public health institutions where there is an extreme lack of resources in the form of products, personnel and expertise. This makes the importance of cost–analysis studies inevitable [6].

The implementation of cost and benefit considerations for medical products and drug therapy has become imperative to efficiently utilize resources. This is especially important in developing countries where the cost of clinical treatments is much higher due to low quality of life as a result of decreased health funding by governments [7]. For this reason, manufacturers and pharmaceutical experts must assess treatment strategies and medical products for both therapeutic effectiveness and cost effectiveness. This is where pharmacoeconomics comes in handy. It provides detailed analysis, including identification, measurement and comparison of the costs and consequences of pharmaceutical and medical products. It helps select the best possible utilization mechanisms for the limited resources available and indicates options accessible for cost-effectiveness and maximizing the benefits of a particular drug or medical product. The objective of this is to evaluate the cost considerations of a drug from its development, formulation and marketing, which means that pharmacoeconomic evaluations cover all the steps from the time the drug is prepared to the time it becomes available to patients [8].

Cost consideration is needed by pharmaceutical companies, healthcare professionals, policymakers and pharmaceutical experts to evaluate the inputs and outcomes of pharmaceutical products, interventions and all types of therapies. It is a discipline that is constantly evolving and evolving with innovation in the healthcare sector. Pharmacoeconomic concepts are fundamental in disciplines such as formulary, cost management for different therapies and reimbursement considerations concerning pharmaceutical and medical drugs and interventions [9].

The Middle East, which comprises 17 South West Asian and North African countries, has announced a wide quality range of healthcare systems. The fleeting economies of Saudi Arabia, United Arab Emirates, Qatar, Kuwait, Israel and Bahrain have excellent healthcare systems with state-of-the-art private and public health facilities, while other Middle Eastern countries such as war-stricken Lebanon, Palestine, Iraq, Iran, Yemen and Syria have very unstable health systems due to the political turmoil in the country and the lack of health funding by erratic governments. This implies that the implementation of pharmacoeconomics in the Middle East region varies in each country with respect to the degree of stability of the healthcare system in that country [10].

The practical implementation depends on numerous factors, including the functioning of the healthcare system, accessibility to quality medications, the effectiveness of the pharmaceutical sector and the funding of healthcare by governments. The efficacy of these factors and many others is directly related to the implementation of pharmacoeconomics in healthcare systems. Many barriers contribute to the defective implementation in all disciplines. It is necessary to overcome these barriers by applying efficacious strategies targeting the pharmaceutical sector and other disciplines related to pharmacoeconomics to optimize the medical system concerning cost considerations of medications and therapies [11].

This review has been conducted to highlight the importance of pharmacoeconomics in the ever-evolving and changing healthcare system in the Middle East. In this article, we have discussed the need of such studies. The various barriers and facilitators to implement and its strategies to overcome them. The level of implementation in different countries of the Middle Eastern region has also been discussed, along with recommendations on how to improve it.

## 2. Materials and Methods

This review was carried out using several research databases to find relevant research studies conducted on the topic ‘Barriers and facilitators of pharmacoeconomics in Middle Eastern countries’. These databases include Google Scholar, MEDLINE, Science Direct and Scopus. The keywords used to find relevant articles include ‘pharmacoeconomics’, ‘Middle East’, ‘barriers’, ‘facilitators’, ‘economic evaluation’, ’pharmacoeconomic implementation’ and ‘Cost analysis’. All the mentioned keywords were searched either alone or in combination using Boolean operators (OR, AND, NOT). All indexing terms in these databases were utilized to modify keywords accordingly so that the most relevant articles could be found. Bibliographies and reference lists of relevant articles were also searched for more applicable studies and titles that were similar to our keywords.

All the databases mentioned above were thoroughly searched to find relevant articles and studies. Study selection was conducted by the analysis of search results. The titles of the resulting studies were checked, followed by abstract analysis and then analysis of the full text of the study to see if it was relevant to our topic or not. Every page of the search results from every database was checked for relevant articles.

All the studies clearly showed information regarding barriers and facilitators of pharmacoeconomics in Middle Eastern countries. All review papers, opinion papers, expert reviews, systematic reviews and original research papers on pharmacoeconomics in any Middle Eastern country were included in the study. However, all articles in any language other than English and articles on pharmacoeconomic studies conducted in any country other than the Middle East were excluded from this study.

## 3. Main Findings

### 3.1. Need for Pharmacoeconomic Studies

Pharmacoeconomics is like a thread that runs through the entire medical and pharmaceutical system and evaluates the cost–benefit relationships of drugs, therapies and interventions through their stages of development, formulation, quality assessment, pricing, marketing and utilization by patients. It is the ultimate science needed to optimize healthcare budgets and make full use of resources at all levels of the healthcare system. As the emphasis on high-quality disease treatment increases, so does the need for cost effectiveness. Health care is becoming increasingly expensive. It is crucial to implement pharmacoeconomic evaluation so that the prices of drugs can be adjusted to levels where patients can easily access them [12].

It is a relatively new discipline and a branch of health economics. Still, it has proven its need and importance with the benefits it brings by practical implementation in a very short time. The need for pharmacoeconomics lies at all levels of the healthcare system, including national governing bodies, industries, hospitals and private and public pharmaceutical sectors [13].

The following points highlight the need for pharmacoeconomics in the healthcare system.

- It provides an assessment of adverse drug reactions, thus aiding efficient pharmacovigilance and reducing negative consequences concerning national health systems.
- It gives a broad insight into a drug or disease’s medical and financial implications.
- Optimizing budget utilization is needed to provide the best possible treatments for a particular disease without putting a heavy cost-related burden on the patients.
- It helps to find alternative treatment plans that are cheaper and more effective for diseases.
- It reveals that newer drugs could be more cost-effective and therapeutically efficient than the overused older ones or Standard of Care (SC) treatment methods.
- It aids in good prescription practice, allowing physicians to prescribe more beneficial and cost-effective medicines that are very favorable for patients.
- It helps in including new drugs in reimbursement and insurance schemes.
- It allows drug price evaluation and highlights the need to fix the price of an existing drug or set the price of a new drug in an optimized way.
- At the industrial level, pharmacoeconomics is required to evaluate the cost of drug formulation as an input and its comparison with the output, i.e., how profitable the drug is in terms of therapeutic efficiency.
- Medication prescribing can be optimized concerning Efficacy, Suitability, Price and Safety (ESPS), with the help of cost-analysis studies.
- It allows effective formulary management by aiding the decision-making process of policies by Pharmacy and Therapeutics (P&Ts) Committees.
- It helps to design health insurance benefits and coverage of treatment costs at the level of private medical systems [1,14,15,16].

A thorough review of current literature available on barriers and facilitators of pharmacoeconomics in Middle Eastern countries led to the identification of barriers and facilitators that have been discussed in detail.

### 3.2. Barriers to Pharmacoeconomic Studies

Despite the great benefits and importance of pharmacoeconomics, this aspect of health economics still faces a large number of challenges in its implementation. Factors related to pharmacoeconomic challenges and barriers may be acceptability and accessibility factors [17]. Acceptability factors include the suitability of using cost-analysis techniques and processes to conduct an outcome evaluation using limited resources and awareness of the importance of pharmacoeconomic application [18]. Other factors related to its challenges include cost considerations, deficiency of experts, availability quality resources and lack of data for formulary decision making [19]. Other hospital-related contributing factors include deficient hospital record keeping policies and lack of attention and time to the evaluation process [20].

**Figure 1:**
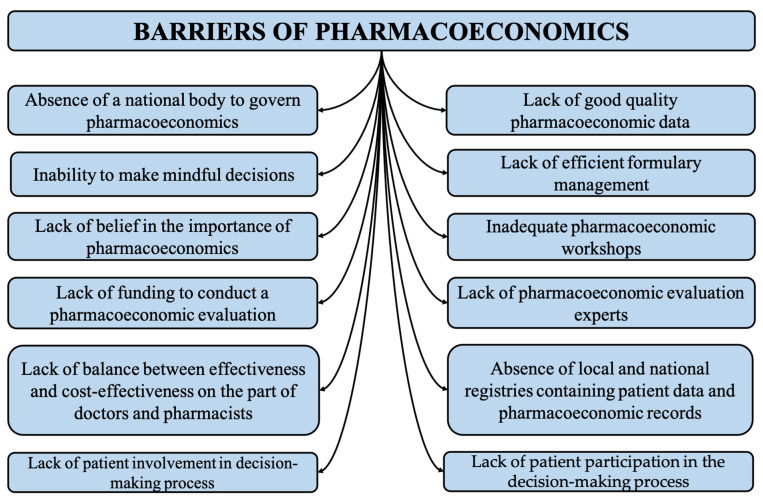
Illustrating major barriers of pharmacoeconomic studies; however, other barriers were discussed in the discussion, such as lack of awareness among audience and public.

Many high-income countries in the Middle East, such as Saudi Arabia, Kuwait, UAE and Qatar, have more developed healthcare systems where the implementation faces fewer challenges and is instead a more straightforward concept as compared to developing, low-income countries, which spend a low percentage of their GDP on healthcare [21]. The following section describes the possible barriers to implementing pharmacoeconomics in different Middle Eastern countries. Figure 1 lists all barriers to pharmacoeconomics.

#### 3.2.1. Absence of a National Body to Govern Pharmacoeconomics

The presence of a national body to govern evaluation studies in a country and study health economics and outcome research is important to keep track of drug performance and cost considerations. Most Middle Eastern countries lack such national non-profit organizations that can perform this task and bring about efficient implementation in the country. The International Society for Pharmacoeconomics and Outcome Research (ISPOR) governs the pharmacoeconomic evaluation of drugs to bring about health improvement and excellence in medication outcome research and has regional chapters and student chapters in many countries worldwide. Among Middle Eastern countries, ISPOR has regional offices in almost all developed countries, but some middle- and low-income countries lack this [22]. This is a huge challenge to implement effective policies and guidelines in these countries. The lack of a governing body creates a leadership gap, and the absence of a system for evaluation and accountability leads to slow and ineffective implementation [23].

To overcome this barrier, governments must develop a national committee or organization responsible for governing all matters related to the implementation of pharmacoeconomics. The responsibilities of this national body should include the organization of local and national workshops, funding for cost-analysis studies, maintenance of national registries for data, and accountability of specialists and experts regarding effective implementation.

#### 3.2.2. Lack of Balance between Effectiveness and Cost-Effectiveness on the Part of Doctors and Pharmacists

It is imperative to evaluate if medications are worth the cost compared to the therapeutic impact they bring about. The absence of this balance between medication effectiveness and cost is a great challenge in implementing high-quality evaluation. When physicians and pharmacists do not consider the cost of the drug compared to therapeutic efficiency, this leads to the unnecessary sale of high-cost medications, which have a therapeutic impact that can be brought about by another medication that is relatively much cheaper [24]. An example of this is the case of the two cardiovascular medications, alteplase and streptokinase. Recent studies on the cost effectiveness of these two drugs have revealed that streptokinase costs ten times less than alteplase while providing the same level of therapeutic efficiency as alteplase. This shows how a lack of balance between the effectiveness and cost-effectiveness of medications can prevent the use and implementation of pharmacoeconomic analyses [25].

To overcome this barrier, physicians and pharmacists need to study and evaluate medications and therapies to determine their ability to achieve the desired therapeutic effect regarding their cost. This can be conducted by properly monitoring the cost–benefit ratio and patient follow-up procedures and the financial concerns of the overall therapy period. Additionally, alternative drugs with the same therapeutic effect can be compared and monitored to rule out high-cost medications.

#### 3.2.3. Absence of Local and National Registries Containing Patient Data and Pharmacoeconomic Records

This barrier to the implementation of pharmacoeconomics is directly related to the absence of an effective healthcare system employing good use of strategies. Pharmacoeconomic registers are essential at both the local and national levels for maintaining records of the costs of medications and therapies, their therapeutic effectiveness data, information on quality adjusted life years (QALY) and health resources. The economic benefit of a healthcare system needs to keep developing and maintaining these registries to document economic evaluations of all kinds and quality of life studies regarding various therapies [26]. In the Middle East, the presence of local data and national records has been observed in high-income countries such as Israel, the UAE and Saudi Arabia, which have better developed health systems compared to low-income countries such as Jordan, Egypt, Yemen, Syria and Palestine where the maintenance of registries is not emphasized due to the lack of expert personnel and health funding [27].

To overcome this barrier, national organizations and specialists must be held responsible for maintaining national registries. Special people should be assigned for this purpose who have the required knowledge of the discipline and maintenance of high-quality records, which can be reasonably utilized for research and evaluation purposes.

#### 3.2.4. Lack of Funding to Conduct a Pharmacoeconomic Evaluation

The governments of developing countries allocate a very small portion of their budget to healthcare, and among that, the budget fixed for pharmacoeconomics is minimal and, in some cases, negligible. This is a great barrier to the methods of conducting such studies. The lack of budgetary funds makes it difficult for pharmaceutical experts and specialists to perform analysis and cost comparisons of different medications and therapies. Lack of stable funding for research for local and federal governments is a major challenge faced by effective health economic evaluation in low-income Middle Eastern countries that are in a developing phase with struggling healthcare systems [28].

Governments need to allocate a specific portion of their health budget for all concerned bodies to overcome this barrier. This can be conducted by making use of national pharmacoeconomic bodies. These funds can be monitored by specialized personnel who can resourcefully distribute the funds at the local and national level to improve the discipline by conducting workshops, aiding researchers and funding evaluation procedures.

#### 3.2.5. Lack of Good Quality Pharmacoeconomic Data

The absence of good-quality data is a crucial barrier to its implementation. When sufficient data on the cost and usage of medications, therapies and patient care are unavailable, it becomes difficult for experts to perform evaluation studies. Studies conducted on the pharmacoeconomic status of the healthcare systems of Jordan, Yemen and Egypt concluded that very little data are available and the only data available are of poor quality, which makes it inadequate for conducting cost analysis studies. Furthermore, local, public and governmental hospitals have unstable systems where recordkeeping is an unimportant task. If it is done, it is done manually. Then the records are lost or not appropriately stored, which causes a lack of efficient data on the cost of therapies, patient hospitalization, medications and various diseases [29,30,31].

The lack of sufficient cost data hinders an investigation of the implications of various healthcare procedures, therapies and treatment methods. The absence of detailed and elaborate cost-of-illness data poses a great challenge in implementing pharmacoeconomic studies. The absence of this cost data can be attributed to a lack of budget-keeping in government hospitals, inefficient recordkeeping of the cost considerations regarding all aspects of the therapies, and failure to maintain high-quality cost data records for individual patients and patients receiving similar treatment therapies or procedures [32].

To overcome this barrier, high-quality patient data must be maintained in all private and public hospitals, and hospital authorities are held accountable for not maintaining such records. For this purpose, a committee must be created that is responsible for the timely checks of hospital records on economic considerations of drugs and diseases.

#### 3.2.6. Inadequate Pharmacoeconomic Workshops

The facilitation of workshops on the part of pharmacoeconomic specialists is essential to inform healthcare professionals of the importance of this aspect of health economics. Healthcare professionals and other members of the healthcare system have minimal knowledge of cost analysis techniques and their importance. Members of committees, physicians and pharmacists need to attend workshops to improve their knowledge of the application. The absence of sufficient workshops and conferences in developing countries can be attributed to lack of funding, low budget, lack of time and lack of experts in this field [33]. An interview study conducted on barriers in Jordan concluded that 98% of respondents who were pharmacists and members of such committees agreed that they needed more workshops to improve their knowledge of this field and enhance their decision making power [29].

To overcome this barrier, governments and national and international bodies should conduct workshops and campaigns on a small and large scale to spread awareness of the importance of this discipline. This can be done by funding these workshops and encouraging experts to participate in such conferences and seminars to educate the healthcare workers on the need for pharmacoeconomics for modern healthcare systems.

#### 3.2.7. Lack of Belief in the Importance of Pharmacoeconomics

Pharmacoeconomics is a young and rather unpopular discipline considered unimportant by many people belonging to the healthcare system. Furthermore, many pharmacy schools in Middle Eastern countries lack education programs. A study conducted on the status of education in the Middle East and North Africa region stated that out of the 176 schools of pharmacy in this region, only 80 schools offer this programs, which accounts for less than 50% of pharmacy schools [10]. This shows the extent of the lack of awareness of the importance of the economic evaluation of pharmaceutical products, which has led to a worse situation of this discipline, especially in low- and middle-income countries.

To overcome this barrier, national pharmaceutical organizations should conduct campaigns. They should educate pharmacy students about the importance of cost analysis studies. Seminars, webinars, presentations, workshops and conferences are the best ways to spread knowledge about the importance of health economics and its applications in pharmacy.

#### 3.2.8. Inability to Make Conscious Decisions

Studies have revealed that the lack of efficient decision making about drug pricing, drug procurement, formulary management, drug distribution and other pharmacoeconomic aspects has compromised this discipline. This occurs when decision makers and experts do not have sufficient knowledge of its importance and applications. Furthermore, many countries lack guidelines for making wise and mindful decisions. They conclude that drugs’ efficiency, safety profile and cost considerations greatly influence the decision-making process. The lack of sufficient data on these aspects of drug therapy is a barrier to good pharmacoeconomic implementation [19].

To overcome this barrier, decision makers and experts need to be more knowledgeable about the rational use of tools and how to use their knowledge in productive ways. Group discussions and the participation of students and researchers can bring about new ideas and innovative approaches, which can greatly lead to rational decision making. Moreover, appropriate pharmacoeconomic guidelines must be made available to experts and decision makers.

#### 3.2.9. Lack of Pharmacoeconomic Evaluation Experts

Despite improving knowledge and awareness of pharmacoeconomics as a discipline, most Middle Eastern countries lack enough specialists in this field who can conduct high-quality evaluation procedures and spread awareness for its need. Experts help make informed decisions about formulary management and drug therapies to obtain sufficient benefits from the available resources. Experts also contribute to studies on pharmacoeconomic evaluation, conduct workshops and explain to students and other members of the healthcare system how it can help improve the cost considerations of medical therapy. Therefore, the lack of pharmacoeconomic evaluation experts is a significant barrier to progress of this field [34].

To overcome this barrier, students must be trained and encouraged to conduct research in this field. They should be provided with funds to conduct research at broader levels. International training sessions should be conducted and coordinated with other national and international organizations to promote the knowledge of pharmacoeconomics and the utilization of budgetary resources, which can significantly help boost the development of discipline.

#### 3.2.10. Lack of Patient Participation in the Decision-Making Process

In the field of pharmacoeconomics, there are several levels of the decision-making process. Firstly, decisions are made at the national or central level where policies are made regarding decisions such as prices and reimbursements of pharmaceutical products and treatment procedures, etc. Secondly, policies and decisions are made at a local or hospital level regarding treatment guidelines or formulary decision making. Lastly, decisions are made at the patient level in healthcare institutions. This level of decision making is highly relevant when it comes to the effective implementation. Unfortunately, in most of the Middle Eastern countries, the role of patient opinion is neglected when it comes to decision making at the patient level. The patient’s perspective on their treatment results and the factors related to the pharmacoeconomics of their entire treatment process can greatly help the decision-making process and help improve policy making and, in turn, better implementation of pharmacoeconomic studies [35,36].

For this purpose, patients should be involved in the decision-making process by interviewing them and allowing them to share their opinions about the pharmacoeconomic aspects of their treatment so that new and better policies can be made that encompass patient concerns as well [35,36].

#### 3.2.11. Lack of Efficient Formulary Management

Formulary management is critical when it comes to an efficient implementation of pharmacoeconomics. Both concepts are interrelated and essential to the progress of the other. It is essential to properly manage hospital formularies considering the safety, efficacy and cost effectiveness of the drugs before adding them to the formularies. This makes formulary data essential for the efficient implementation of pharmacoeconomics [37]. 

Hospital formularies must be appropriately managed and checked by national pharmacoeconomic governing bodies to overcome this barrier. Furthermore, appropriate guidelines must be provided on which drugs to add to the formularies and how efficient formulary management can maintain drug and patient records.

#### 3.2.12. Lack of Public Awareness Regarding the Importance of Pharmacoeconomics

The applications of pharmacoeconomics and their positive impact on a healthcare system cannot be achieved and benefitted from unless there is a sound awareness of its importance among the audience of researchers, students, decision makers and healthcare practitioners. The impact of pharmacoeconomics on the importance of economic evaluation of healthcare and patient-reported outcomes of treatments and medicines and the quality of life of patients is greatly influenced by the degree of awareness regarding the cost consideration of medications and the economic aspects of pharmaceuticals. Lack of awareness is one of the main barriers to the effective implementation of pharmacoeconomic studies and its positive impact on economic policy making [9,38].

To overcome this barrier, there is a need to conduct workshops, seminars, national and international guidelines, and awareness programs to educate the general public, students, researchers and decision makers. The government can take action in this regard by disseminating knowledge and pharmacoeconomic guidelines in educational institutions, group discussions and research panels. 

In Table 1, all the barriers mentioned in this review were presented, and strategies how to overcome these barriers are presented.

### 3.3. Facilitators of Pharmacoeconomic Studies

**Figure 2:**
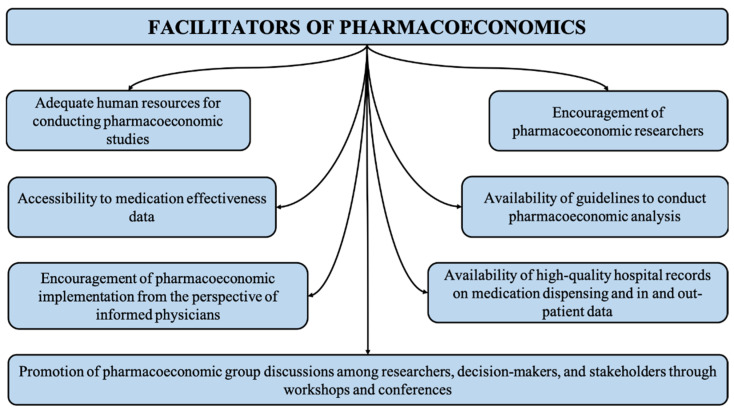
Illustrating facilitators of pharmacoeconomic studies.

As there are many barriers to the implementation of pharmacoeconomics, there are also many facilitators that promote the effective use and application in healthcare systems. These facilitators are related to the availability of patient records, direct and indirect cost data and applicable guidelines, pharmacoeconomic experts, awareness of the discipline and decision making (Figure 2). In the following section, we describe the potential facilitators for implementing pharmacoeconomics in Middle Eastern countries.

#### 3.3.1. Accessibility to Medication Effectiveness Data

To conduct good-quality pharmacoeconomic studies, experts, researchers and specialists in this field need to access the data on the effectiveness and cost considerations of drugs and therapies and the financial consequences of diseases. These data allow researchers to effectively implement them for decision making and mindful analysis of drug therapies by balancing their therapeutic efficiency and cost-effectiveness so that treatments can be optimized in favor of both patients and healthcare professionals. Furthermore, the availability of medication effectiveness data helps rule out unproductive high-cost medications and therapies and brings forward cheaper and more beneficial alternatives. Thus, this factor is a great facilitator in the effective implementation of pharmacoeconomic studies [39].

#### 3.3.2. Availability of Guidelines for Conducting Pharmacoeconomic Analysis

To effectively implement pharmacoeconomic studies in a healthcare system, proper quality guidelines must be available for researchers and experts. Guidelines allow effective use of resources to collect data on medications and therapies and cost analyses of diseases and treatment strategies so that medical therapy can be optimized and budget friendly. ISPOR has developed guidelines that have been made available to most Middle Eastern countries through the regional and student chapters of the organization. These guidelines provide suitable methods for the implementation of studies and the cost analysis of drugs and diseases [40].

#### 3.3.3. Encouragement of Pharmacoeconomic Researchers

Researchers and experts are the people who conduct pharmacoeconomic studies. Their encouragement is necessary for the effective implementation of pharmacoeconomics. The best steps in this regard are to provide them with funds to conduct workshops and to conduct awareness programs on the importance of this discipline in the field of healthcare. Effective implementation can be carried out by aiding experts in conducting scientific research and finding ways to improve analysis methods. In this way, medication efficiency can be resourcefully optimized, and the expertise of pharmacoeconomic specialists can be utilized to reduce the cost burden of diseases and their therapy. Therefore, the encouragement of researchers serves as a great facilitator in the implementation of this discipline [20].

#### 3.3.4. Adequate Human Resources for Conducting Pharmacoeconomic Studies

The availability of human resources to conduct research is crucial for the successful application of pharmacoeconomic studies. No analysis can be conducted efficiently unless an adequate number of experts and decision makers in that discipline are accessible. The number of experts in this field can be improved by promoting education in schools of pharmacy and other medical sciences and spreading awareness of the discipline through workshops and conferences. Furthermore, the presence of experts encourages newcomers to the field and paves the way for improvement and innovation through mindful decision making and group efforts. Training programs and workshops regarding implementation should train professionals to make a well-equipped human resource available for conducting pharmacoeconomic studies [20]. Experts should be associated with clinics, local hospitals, general hospitals and district hospitals. They should be responsible for maintaining records and ensuring the availability of adequate cost data. Encouragement of human resources to conduct research and improve their publication skills greatly benefits the implementation process of pharmacoeconomic studies and the dissemination of research findings [41]. 

#### 3.3.5. Promotion of Pharmacoeconomic Group Discussions among Researchers, Decision-Makers and Stakeholders through Workshops and Conferences

Pharmacoeconomic workshops and conferences are the best way of joining experts and specialists in the field with decision makers and stakeholders to work together for improvements in the discipline. Arrangement of such workshops and seminars on local and national levels greatly helps boost knowledge of pharmacoeconomics among students and researchers. It brings about new ideas and ways to bring innovation to the healthcare system by implementing pharmacoeconomic tools and making full use of financial resources and budget management by creating a balance between drug costs and results [42].

#### 3.3.6. Availability of High-Quality Hospital Records on Medication Dispensing and In- and Out-Patient Data 

Medical records and patient data are the sources from which information is taken and submitted for pharmacoeconomic analysis. Therefore, adequate data must be available on the use of drugs and therapies among patients for different diseases, their cost considerations and other budgetary aspects. The availability of high-quality records and sufficient information in this respect and easy access to it is crucial for the effective implementation of pharmacoeconomic evaluation in a healthcare system. These data can be made available by maintaining national and local registers and records and assigning specific people. Thus, this factor can be considered one of the main facilitators of successful pharmacoeconomic implementation [43].

#### 3.3.7. Encouragement of Pharmacoeconomic Implementation from the Perspective of Informed Physicians

Physicians who have a positive attitude towards the economic aspects of healthcare procedures play a vital role in the effective implementation of pharmacoeconomic studies. They aid the decision-making process and establishment of guidelines for conducting research. Furthermore, physicians play a vital role in making formulary decisions, significantly impacting economic evaluation studies. The critical role of physicians in the healthcare department is prescribing. Their prescribing patterns greatly influence the efficiency of research and allow researchers to investigate the most prescribed medications and treatment procedures, as well as their economic impact and cost considerations. Therefore, the availability of informed physicians is a significant facilitator in the effective implementation of pharmacoeconomic research [44].

## 4. Discussion of Pharmacoeconomic Studies with Middle Eastern Countries

In this study, the current literature available on barriers and facilitators to pharmacoeconomic studies in Middle Eastern countries was reviewed. The healthcare systems of Middle Eastern countries were evaluated to verify the status of the pharmacoeconomics in them. 

The Middle Eastern countries are very similar in their sociocultural aspects. However, their economies vary greatly and their ways of meeting the healthcare needs of their citizens are also very different [45]. In addition to the highly flourishing economies of the UAE, Qatar, Saudi Arabia, Kuwait, Bahrain, Oman, Cyprus and Israel, which are classified as high-income countries according to the World Bank classification system, many low-income countries in the Middle East are stricken by war and have withering economies and unstable systems. These include Yemen and Syria. Other lower–middle income countries include Palestine, Iran and Egypt. Upper–middle income countries include Iraq, Lebanon, Jordan and Turkey [46].

Saudi Arabia, one of the largest countries in the Middle East, has a large consumer market. It has a gross domestic product (GDP) of USD 23,762.42 per capita. According to the data obtained from the World Health Organization database, Saudi Arabia spends about 5.69% of its GDP on healthcare [47]. Despite having a flourishing economy, the Saudi Arabian healthcare system faces many challenges, such as the high prices of drugs due to the high cost of drug manufacturing, which comes from stringent regulatory measures faced by manufacturers. Moreover, preferences for branded drugs in place of generics and easy availability of drugs from the pharmacy without a prescription contribute to these challenges. Therefore, the Saudi Arabian system eagerly requires the implementation of pharmacoeconomics so that drug utilization in the country can be optimized. Pharmacoeconomic analysis techniques can answer drug-related questions such as, are they worth the money? Does this treatment have good value for money? How can budget utilization be optimized for pharmacoeconomics [48]? 

Similarly to Saudi Arabia, UAE, Israel, Kuwait and Qatar are also high-income countries in the Middle East. The GDP per capita is USD 43,103.34, USD 43,610.52, USD 50,805.46 and USD 32,373.25 for UAE, Israel, Qatar and Kuwait, respectively. Current healthcare expenditures are 4.2%, 7.5%, 5% and 2.5% of GDP for the UAE, Israel, Kuwait and Qatar, respectively [47]. With such good resources and developed economies, these high-income countries have innovated their healthcare systems. Several studies have been carried out to assess the health economic and pharmacoeconomic situation in these countries and the potential barriers to their implementation.

El Jardali et al. conducted a study to assess the status in Eastern Mediterranean countries. They suggested that the absence of coordination and group work between pharmacoeconomic researchers and health policymakers is a significant barrier to the implementation [49]. Almazrou et al. reported that researchers face difficulty using pharmacoeconomic tools due to a lack of expertise on evaluation outcomes such as QALYs. Furthermore, there is no national governing body solely dedicated to implementing pharmacoeconomics at the local and national levels [42].

Many studies have been conducted worldwide [48,49,50] that assess the potential barriers and facilitators to implementing pharmacoeconomics. These are comparable to those discussed in our study found in studies conducted in the Middle East. Luz et al. conducted a study on assessing challenges faced in the implementation, use and reporting of pharmacoeconomic evaluation procedures. The biggest challenge was the lack of valuable, relevant data [50]. This is consistent with the findings of Alefan et al., who conducted a study to assess barriers and facilitators to implement pharmacoeconomics in Jordan. They found that lack of adequate patient data and its cost considerations, the absence of computerized health reporting systems, lack of funds from the government, inadequate formulary management, the lack of experts and the absence of awareness among pharmacy students regarding the importance of pharmacoeconomics [29]. 

The countries in the Middle East that fall under the lower to middle income level of classification, include Yemen, Syria, Iran, Iraq, Jordan, Egypt, Lebanon and Palestine. They have very limited healthcare sectors with minimal pharmacoeconomic implementation. The GDP per capita is USD 824.12, USD 2032.62, USD 3239.73, USD 3547.87, USD 3598.48, USD 4282.77, USD 4891 and USD 5523.08 for Yemen, Syria, Palestine, Egypt, Jordan, Iran, Lebanon and Iraq, respectively, as per the data obtained from the database of the World Bank [51]. The healthcare expenditure for Yemen, Syria, Iran, Iraq, Jordan, Egypt, Lebanon and Palestine is 4.9%, 3.57%, 8.6%, 4.1%, 8.6%, 4.75%, 8.4% and 13.7% of the GDP, respectively [47]. This accounts for significantly less expenditure on healthcare. Based on these data, the implementation of pharmacoeconomics in these low-income countries is minimal. These countries have small budgets and inadequate resources to deal with the heavy load of various diseases. The implementation in these countries is challenged by numerous factors, among which the scarcity of resources is the largest. Furthermore, the lack of researchers and awareness among policymakers hinders the use of pharmacoeconomic tools in the healthcare system. Given the low-income countries’ current situation, applying pharmacoeconomics is a tedious task due to the unstable healthcare systems and inadequate pharmacy education. Furthermore, the lack of patient records and national registries also contributes to the absence of research in the state [52].

Al Harakeh and his coworkers assess the implementation of pharmacoeconomics and health technology assessment in Lebanon. They found that the lack of funds is the main barrier to implementing pharmacoeconomics at the state level. A major facilitator was the availability of researchers to assess pharmacoeconomics and the application of analytical tools to assess the cost considerations of medications and treatment strategies [53].

Cheraghali conducted a study to evaluate the implications in the Iranian healthcare system. He reported that pharmacoeconomics is implemented in the system by promoting education in pharmacy schools and that there is a minimal role for pharmaceutical industries in this regard. The Iranian pharmaceutical sector has limited resources in terms of funds and human experts, which poses a significant challenge in applying an effective pharmacoeconomic evaluation in the system [54].

Education in pharmacoeconomics is one of the key factors that can improve the status in the Middle East. A study conducted by Farid and Baines on the status of pharmacoeconomics education in Middle Eastern countries showed that there are 80 schools of pharmacy in the Middle East, which accounts for only 45% of the total number of high schools in the region. Of these 85 schools, 65 offer pharmacoeconomics education at the postgraduate level, 8 at the graduate level and 7 at both levels [10]. The highest number of pharmacy schools are present in Egypt, i.e., 24 and only 7 of them offer courses [55]. Next in line is Saudi Arabia, which has 16 schools of pharmacy, of which only three offer pharmacoeconomics courses. Iraq has 14 schools of pharmacy, of which 5 offer pharmacoeconomic courses and 2 claim that they will offer them in the near future [56]. This shows the weak state of pharmacoeconomic education in the Middle East. 

Pharmacoeconomics is a very important deciding factor in pharmaceutical decision making, rational drug usage, allocation of health resources and health insurance policies. Thus, promoting pharmacoeconomic education in schools is imperative to benefit from its advantages. This can greatly help in improving the economic impacts of pharmaceutical procedures and can bring about a remarkably positive change in the quality of life of the general public by the implication of innovative ideas of pharmacoeconomic students and researchers. For this purpose, it is necessary to emphasize the addition of pharmacoeconomic courses in pharmacy schools and both the professional and graduate levels. International conferences, workshops and training should be conducted to spread awareness regarding the importance of pharmacoeconomic education and its impact on the long-term pharmaceutical picture of a country [57,58].

Another very important pharmaceutical aspect associated with pharmacoeconomics is polypharmacy. It refers to the use of at least five drugs daily to treat multiple morbidities. This is usually common in older adults who suffer from multiple diseases such as diabetes, hypertension and cardiovascular disease or in younger populations at risk. Polypharmacy is linked with considerable economic implications in the pharmaceutical sector. A study by Aseel et al. on polypharmacy in Saudi geriatric patients revealed that 55% of their study participants were involved in polypharmacy and this causes a great toll on the budgetary concerns of the pharmaceutical sector [59]. The cost of multiple medications is huge and it can cause an approximate 30% increase in the medical costs of a patient [60]. Abu Farha et al. conducted a study in Jordanian hospitals to assess the prevalence and predictors of polypharmacy. They found that polypharmacy is expensive and only people with a high-income status can afford to take multiple medications and adhere to their treatment, which is mostly not covered by insurance plans due to high costs [61].

Bagher et al. conducted a study to evaluate the knowledge, perception and confidence of healthcare professionals about pharmacogenetics and reported that, on average, healthcare professionals have low knowledge about PGx, but positive perception about PGx testing and its implications. In self-confidence, they reported a moderate level when using PGx testing. The authors reported that people who had completed postgraduate studies had a higher level of knowledge compared to others [62]. In another study, the author states that costs of pharmaceuticals increased in most countries, and alarming caused healthcare professionals to adopt cost-conscious behavior when prescribing medication to the patients. The study concluded that physicians agreed to adopt cost-conscious behavior as they had enough knowledge of medicines, but they had less knowledge of medication costs [63]. More such empirical/observational studies were needed to find out the results from the real world, and policies can be made for effective implementation of pharmacoeconomics. Numerous barriers of varying nature face the effective implementation of pharmacoeconomics in Middle Eastern countries. However, many facilitators can contribute to the progress and awareness of this discipline in healthcare systems. Further research in this field can help optimize the healthcare system and efficiently use pharmacoeconomic tools.

## 5. Recommendations for Implementation

With the rise in healthcare costs and the ever-increasing demand for medications due to the prevalence of diseases, the need for pharmacoeconomics in all countries has become crucial. There is a need to develop ways and methods to promote the effective implementation of pharmacoeconomics in a state’s healthcare system. In this respect, the following are specific recommendations by which the implementation of pharmacoeconomics in a country can be improved:

- The availability of suitable guidelines for formulary management, pharmacoeconomic research and the use of pharmacoeconomic tools must be ensured at the local and national levels. These guidelines should also compare the therapeutic efficiency of drugs and their cost-effectiveness compared to similar drugs [64].
- Effective application of pharmacoeconomic studies must be done in pharmacy schools and other health schools implementing pharmaceutical studies in their curricula. Students should be aware of the importance of pharmacoeconomics for healthcare systems. Workshops and seminars can do this in institutes to develop the interest of the discipline and bring forward more specialists and experts in the field to carry out research and apply pharmacoeconomic tools to healthcare data [65].
- Keeping efficient health data records must be compulsory for all hospitals at the government and private levels. This is highly important because the availability of high-quality medication data is the biggest facilitator of the implementation of pharmacoeconomics. Governments should establish minimum data set requirements and form committees that evaluate medical records and data sets and hold the authorities responsible for poor health records [42].
- Pharmacoeconomic workshops and awareness campaigns should be conducted at the local and national levels, which can also be extended to international collaborations if funds are available. In this way, the importance of pharmacoeconomics can be communicated to young researchers and healthcare professionals.
- There should be effective coordination between health policymakers and researchers so that discrepancies in the system can be conveyed and new and better policies can be devised to overcome barriers to implementing pharmacoeconomics.
- States should establish and develop national organizations and agencies responsible for governing pharmacoeconomic implementation and making all the necessary decisions and actions necessary to make good use of pharmacoeconomic analyses in healthcare systems.
- Recordkeeping of all medical data should be maintained, including the cost of treatment, duration of treatment, lifestyle impacts of a disease and its therapy, epidemiological and demographical medical data and patterns of disease occurrence and clinical practice of various therapies [66].
- National and local pharmacoeconomic organizations should publish the actual costs of medication and healthcare services for specific therapies and medical procedures to set up a standard for pharmacoeconomic studies and aid pharmacoeconomic researchers by providing high-quality cost data.

## 6. Conclusions

With the constant rise in healthcare costs, the need for pharmacoeconomics has been growing. The Middle East, a region of various economies, is ideal for conducting pharmacoeconomic research. It constitutes both high- and low-income countries, thus providing a wide range of healthcare systems to evaluate. A review of the literature has led to the conclusion that the lack of adequate medical data and its accessibility, the absence of pharmacoeconomic experts, the lack of awareness of the importance of pharmacoeconomics, the absence of national governing bodies, inefficient recordkeeping and inadequate formulary management are significant barriers to pharmacoeconomics implementation. These barriers can be resolved by the formation of national pharmacoeconomic regulatory authorities, the maintenance of patient treatment data, efficient formulary decision making, the encouragement of pharmacoeconomic researchers and the availability of guidelines for pharmacoeconomic studies. In contrast, the most prominent facilitators observed in the literature were the availability of sufficient funds in high-income countries, the accessibility to drug effectiveness data, the encouragement of pharmacoeconomic experts and researchers, the availability of pharmacoeconomic guidelines and the promotion of group discussions, workshops and awareness campaigns to improve knowledge of the discipline among students. There is still a great need for improvement so that states can effectively benefit from the tools of pharmacoeconomic analysis and use their health resources. In this sense, they should develop national governing bodies to evaluate and implement pharmacoeconomics at the local and state levels and bring about innovation in the field through further research and development incorporating all sectors of pharmacy and pharmaceutics. The data presented in this research can further be extended in future studies to cover the various domains of pharmacoeconomics including cost-minimization analysis, cost-effectiveness analysis and cost-benefit analysis and their applications within the healthcare sectors of Middle Eastern countries.

## Figures and Tables

**Table 1:** Barriers to pharmacoeconomics and their counter strategies to overcome.

Barrier	Strategies to Overcome	Reference
Absence of a national body to govern pharmacoeconomics	Development of a national regulatory committee on pharmacoeconomics	[23]
Lack of balance between effectiveness and cost effectiveness on the part of doctors and pharmacists	Proper monitoring and comparison of the cost–benefit ratio of drugs and patient follow-up procedures	[25]
Absence of local and national registries containing patient data and pharmacoeconomic records	Allocation of specialized personnel for maintaining local and national registries and conducting proper accountability	[27]
Lack of funding to conduct a pharmacoeconomic evaluation	Allocation of a specific health budget by governments to conduct pharmacoeconomic research and evaluation	[28]
Lack of good quality pharmacoeconomic data	Maintenance of high-quality patient records in hospitals	[31]
Inadequate Pharmacoeconomic Workshops	Organization of pharmacoeconomic workshops at a national and international level, encouragement of experts to participate in workshops.	[33]
Lack of belief in the importance of pharmacoeconomics	Awareness campaigns for young students and researchers to disseminate information about the importance of pharmacoeconomics	[10]
Inability to make conscious decisions	Education of decision makers and experts on new approaches arising in the field and encouragement of group discussions with researchers	[19]
Lack of pharmacoeconomic evaluation experts	Encouragement of young researchers to participate in pharmacoeconomic studies, allocation of funds for experts	[34]
Lack of efficient formulary management	Establishment of appropriate guidelines for formularies that frequently investigate the quality of hospital formularies by national governing bodies	[35]
Lack of patient participation in the decision-making process	Encouragement of patients to participate in decision making through interviews and opinion sharing	[2]
Lack of public awareness regarding the importance of pharmacoeconomics	Workshops, training programs and seminars to spread knowledge of pharmacoeconomics among decision makers, students and researchers	[38]

## Data Availability

Not applicable.
